# Data on recovery rates and external morphologies of zircon grains from mechanical and electrical pulverization of rock samples

**DOI:** 10.1016/j.dib.2018.06.016

**Published:** 2018-06-18

**Authors:** Mami Takehara, Kenji Horie, Tomokazu Hokada, Shoichi Kiyokawa

**Affiliations:** a National Institute of Polar Research, 10-3, Midori-cho, Tachikawa-shi, Tokyo 190-8518, Japan; b Department of Polar Sciences, The Graduate University for Advanced Studies (SOKENDAI), 10-3, Midori-cho, Tachikawa-shi, Tokyo 190-8518, Japan; c Department of Earth and Planetary Sciences, Kyushu University, 744 Motooka, Nishi-ku, Fukuoka, 819-0395 Japan

## Abstract

In this data article, we provide information on the recovery rate and scanning electron microscope (SEM) images of the external morphology of zircon grains separated from two rock samples (AS3 and TEMORA 2) using both mechanical and electrical pulverization systems. The data in this article are related to the research article entitled “New insight into disturbance of U-Pb and trace-element systems in hydrothermally altered zircon via SHRIMP analyses of zircon from the Duluth Gabbro” (Takehara et al., 2018) [1]. Zircons from these two rock samples are widely used as reference materials for U–Pb dating by micro-beam techniques. Rock samples with nearly equal weights were pulverized by both methods, and the recovered zircon grains were then concentrated using conventional mineral-separation methods. Weights of the products at each step in the mineral separation process were measured, and finally the recovery rates of the heavy and non-magnetic minerals, including zircon, were calculated.

**Specifications Table**

**Table:** 

Subject area	*Earth and Planetary Sciences*
More specific subject area	*Mineralogy*
Type of data	*Tables and Figures*
How data was acquired	*High voltage pulse power equipment (SELFRAG Lab), Scanning electron microscope (SEM; JEOL JSM-5900LV), Field emission-scanning electron microprobe (FE-SEM; JEOL JSM-7100F) and Electronic balance (ASP413)*
Data format	*Raw and calculated*
Experimental factors	*The rock samples were cut into a suitable size and shape for each pulverization method by using a rock cutter. They were then cleaned in an ultrasonic bath and dried in an 80 °C oven.*
Experimental features	Rock samples (TEMORA 2 and AS3), with nearly equal weights, were pulverized by two methods: mechanical pulverization (using a stamp mill) and electrical pulverization (SELFRAG Lab). Each crushed rock sample was separated by conventional mineral separation methods, and recovery rates were calculated based on the weights of the products of mineral separation. External morphologies of zircon grains collected from each crushed and separated rock sample were observed by SEM.
Data source location	*TEMORA2: Middledale Gabbroic Diorite in the Lachlan Fold Belt of southeastern Australia*
	*AS3: Anorthosite Series (AS3) in the Duluth complex, Minnesota, U.S.A.*
Data accessibility	*Data are within this article.*
Related research article	*M. Takehara, K. Horie, T. Hokada, S. Kiyokawa, New insight into disturbance of UPb and trace-element systems in hydrothermally altered zircon via SHRIMP analyses of zircon from the Duluth Gabbro, Chem. Geol. 484 (2018) 168–178.*[1]

**Value of the Data**

● This data article provides important information about the impact of pulverization methods on zircon recovery rates in a rock sample, since enhancement of the zircon recovery rates is important for accurate micro-beam analysis, especially if polychromic zircon populations are to be quantitatively compared.
● This data article indicates that the recovery rate of the reference zircons for U–Pb geochronology using micro-beam techniques, by electrical and mechanical pulverization methods, which is an essential information for practical mineral separation of zircon for U–Pb geochronology using micro-beam techniques
● This data article shows the external morphologies of zircon grains separated by each pulverization method, and provides information about how the pulverization of rock samples break or preserve the external morphologies of zircon grains.

## 1 Data

### 1.1 Recovery rate of zircon grains

Recovery rates were estimated from the products at each step in the mineral separation of the rock samples. In this article, we focus on two pulverization methods for rock samples in the mineral separation process: mechanical (stamp mill) and electrical (SELFRAG Lab) pulverization. The SELFRAG Lab system is a commercial lab-sized machine developed by SELFRAG AG that pulverizes materials using the pulse power of high voltage discharge (e.g., [2]). The equipment pulverizes materials in dielectric water (i.e., ion-exchanged water) by high-voltage discharge (90–200 kV) and liberates material along natural boundaries (i.e., grain boundaries in rocks). Therefore, the equipment has a high potential to minimize the loss of mineral grains and to keep the original shapes of mineral grains during pulverization. The mechanism of the equipment is described in more detail on the SELFRAG AG official website (http://www.selfrag.com/index.php).

**Fig. 1:**
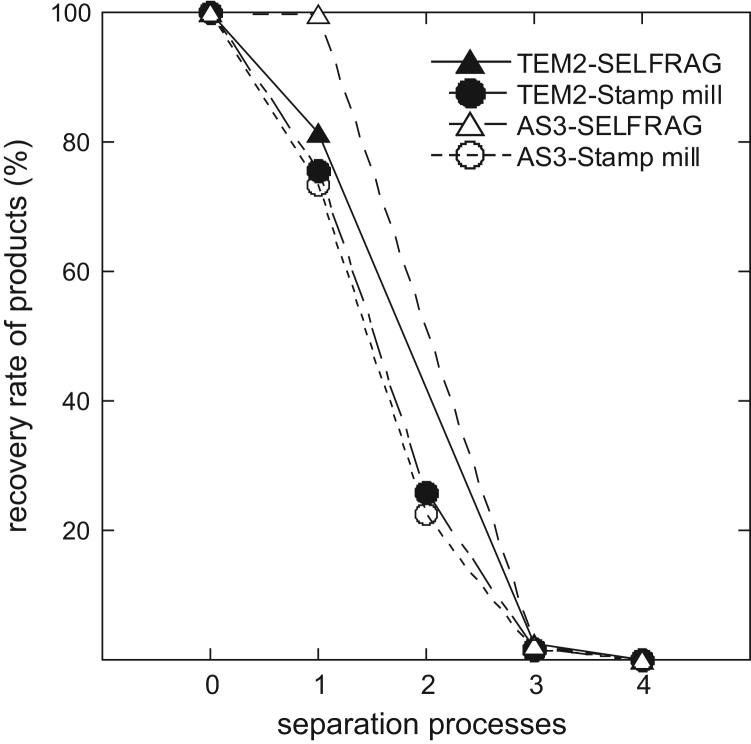
Recovery rates of the products in each separation process. The numbers for the separation processes (x-axis) correspond to those in Table 1: 0. original rock sample (100%), 1. pulverization, 2. elutriation (rinsing with water), 3. heavy liquid separation, 4. magnetic separation. The recovery rate of the products is described in the main text.

We show the weights of the products for each separation process in Table 1. In this data article, the recovery rate is defined as the ratio of the weight of the product for each separation process to the weight of the starting material. The recovery rates of TEMORA 2 and AS3 zircons are shown in Table 1 and plotted in Fig. 1. The recovery rates for each separation process are described more fully in the next section (Experimental Design, Materials, and Methods).

**Table 1:** The weight of product through each separation process.

	0	1	2	3	4
	Rock sample	Pulverization	Elutriation (rinsing with water)	Heavy liquid separation	Magnetic separation
***TEMORA 2***					
SELFRAG	378.5	307.9	–a	9.3	0.0361
Stamp mill	383.0	290.0	98.9	6.6	0.0268
					
***AS3***					
SELFRAG	422.1	421.4	–a	9.6	0.147
Stamp mill	421.7	309.1	94.6	5.7	0.070

a In the separation process after SELFRAG pulverization, rock samples were not elutriated by using a tall beaker and the weights of products were not measured.

### 1.2 SEM images of the external morphologies of zircon grains

**Fig. 2:**
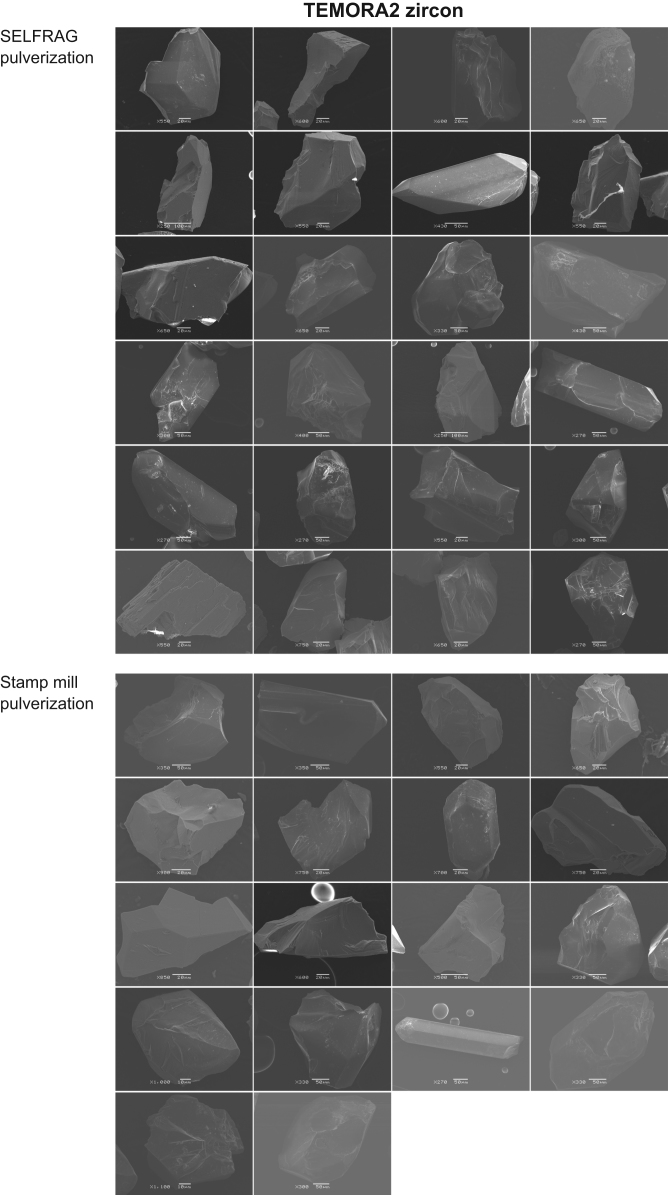
SEM images of the external morphologies of TEMORA 2 zircons. The upper part of the figure shows zircons separated through SELFRAG pulverization, and the lower part shows zircons separated through stamp mill pulverization.

**Fig. 3:**
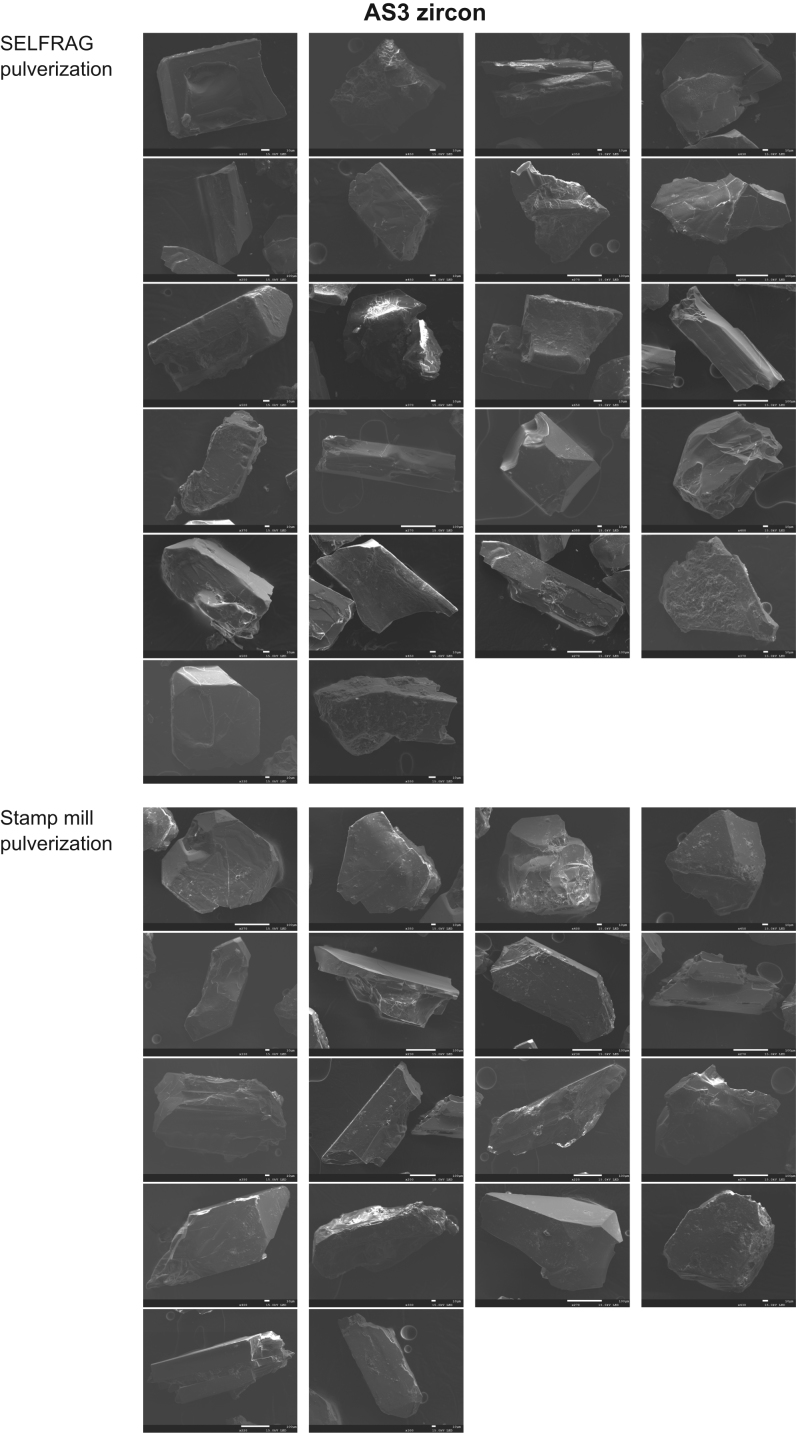
SEM images of the external morphologies of AS3 zircons. The upper part of the figure shows zircons separated through SELFRAG pulverization, and the lower part shows zircons separated through stamp mill pulverization.

Zircon grains separated by both pulverization methods were handpicked, and their external morphologies were observed using a scanning electron microscope (SEM: JEOL JSM-5900LV) and a field emission SEM (FE-SEM: JEOL JSM-7100F). TEMORA 2 zircon grains were observed using SEM (Fig. 2) and AS3 zircon grains were observed using FE-SEM (Fig. 3).

## 2 Experimental design, materials, and methods

### 2.1 Materials

TEMORA 2 zircons and AS3 zircons are the reference materials used for U–Pb geochronology by micro-beam techniques such as secondary ion mass spectrometry (SIMS) and laser ablation inductively coupled mass spectrometry (LA–ICP–MS). The host rocks of TEMORA 2 zircons and AS3 zircons are the Middledale gabbroic diorite in the Lachlan Fold Belt of southeastern Australia [3], [4] and the Duluth gabbroic anorthosite from Minnesota, USA. [5], respectively.

### 2.2 Mineral separation

**Fig. 4:**
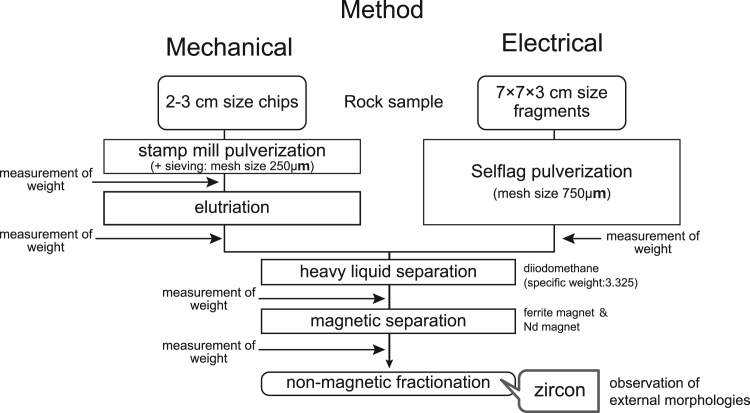
Scheme of the mineral separation process for zircon.

The rock samples (TEMORA 2 and AS3) were cut into fragments of nearly equal weights for both pulverization methods. The weights of the rock fragments for both samples are reported in Table 1. The rock fragments were pulverized by using a stamp mill in the mechanical pulverizing method and by using a SELFRAG Lab system in the electrical pulverizing method. Minerals in the pulverized samples were then separated by conventional mineral separation processes, including elutriation, heavy liquid separation, and magnetic separation (Fig. 4).

#### 2.2.1 Pulverization

For pulverization by the stamp mill, a 383.0 g fragment of TEMORA 2 and a 400.0 g fragment of AS3 were cut into 2–3 cm chips. The detailed process is as follows.

i. Every 100 g of the rock chips was pulverized by the stamp mill for about 20 minutes.
ii. The crushed rock samples were sieved by using a 250-μm sieve, and products finer than 250 μm were collected.
iii. The residual crushed rock sample in the 250-μm sieve was collected and pulverized by the stamp mill again for about 20 minutes.
iv. Steps i to iii were repeated until the amount of residual sample (coarser than 250 μm) stopped decreasing.

Finally, the total amount of products finer than 250 μm collected in step ii was weighed by using an electronic balance. The weight of each rock sample is reported in Table 1.

In the electrical pulverization method, we pulverized a 378.5 g fragment of TEMORA 2 and 422.1 g fragment of AS3, both 7 cm×7 cm×3 cm, using the SELFRAG Lab system. Pulverization of the rock fragments was performed in the following steps.

i. The rock fragment was put into a SELFRAG process vessel filled with ion-exchanged water.
ii. The rock fragment was pulverized by electrical discharge. The configuration of the SELFRAG pulverization system is described in Table 2.

**Table 2:** The configuration of SELFRAG.

Rock sample	TEMORA 2	AS3
Gap (mm) between electrodes	17	20
Total number of discharge pulse	1000	1000
Frequency (Hz) of discharge pulse	3	3
Voltage (kV)	140	120
iii. After pulverization, products passing through a 710-μm sieve with 45% transparency were collected, and suspended fine particles in the water were disposed of.

Finally, the total amount of products collected in step iii was weighed by using an electronic balance. The weight of each rock sample is compiled in Table 1.

#### 2.2.2 Elutriation

After pulverization by the stamp mill, the finer products were separated based on the specific gravity of each mineral in water. Each finer product was put into a tall glass beaker with water and mixed thoroughly. Suspended fine particles mainly composed of clay minerals were disposed of. This process was repeated until the suspended fine particles disappeared almost completely. The finer products remaining in the beaker were collected and weighed by using the electronic balance. The weight of each rock sample is described in Table 1.

In the case of SELFRAG pulverization, the finer products were not elutriated because suspended fine particles in water were already disposed of during the pulverization process.

#### 2.2.3 Heavy liquid separation

After elutriation, the products were separated based on the specific gravity of each mineral in the heavy liquid, diiodomethane (specific gravity: 3.325). Minerals heavier than diiodomethane that sank to the bottom of the separating funnel were collected. The sinking mineral fractions were rinsed carefully with acetone and weighed by using the electronic balance. The weights of the heavy mineral fractions are compiled in Table 1.

#### 2.2.4 Magnetic separation

After heavy liquid separation, the heavy mineral fractions were separated by hand using a Nd-magnet. The non-magnetic fractions, which remained after removing the magnetic fractions, were collected and weighed by using the electronic balance. The weight of the non-magnetic fractions is compiled in Table 1. The non-magnetic fractions contain substantial amounts of zircon grains.

### 2.3 SEM observation of the external morphologies of zircon grains

External morphologies of zircon grains handpicked from the non-magnetic fractions were observed by SEM and FE-SEM. The zircon grains were mounted on a piece of double-sided tape and coated with carbon for observation. TEMORA 2 zircons and AS3 zircons were observed using SEM and FE-SEM, respectively. Configuration of the SEM observation conditions is as follows: accelerating voltage is 15 kV, probe current is about 0.3 nA, and working distance is 10 mm. Configuration of the FE-SEM is as follows: accelerating voltage is 15 kV, probe current is about 10.4 nA, and working distance is about 9.48 mm.
