# Supplementary material for: Data on recovery rates and external morphologies of zircon grains from mechanical and electrical pulverization of rock samples
Source: Data Brief. 2018 Jun 18;19:1537–44. doi: 10.1016/j.dib.2018.06.016 (PMC6141137; doi:10.1016/j.dib.2018.06.016)
Supplement: Supplementary file 1 — Supplementary material [file mmc1.pdf]

Conflict of Interest and Authorship Conformation Form

Please check the following as appropriate:

- o All authors have participated in (a) conception and design, or analysis and interpretation of the data; (b) drafting the article or revising it critically for important intellectual content; and (c) approval of the final version.

- o This manuscript has not been submitted to, nor is under review at, another journal or other publishing venue.

- o The authors have no affiliation with any organization with a direct or indirect financial interest in the subject matter discussed in the manuscript

- o The following authors have affiliations with organizations with direct or indirect financial interest in the subject matter discussed in the manuscript:

Author’s name	Affiliation
Mami TAKEHARA	National Institute of Polar Research
Kenji HORIE	National Institute of Polar Research
Tomokazu HOKADA	National Institute of Polar Research
Shoichi KIYOKAWA	Kyushu University
